# Assessing Age-Friendliness in Higher Education: Introducing the Inventory and Campus Climate Surveys

**DOI:** 10.1093/geroni/igaa057.1738

**Published:** 2020-12-16

**Authors:** Nina Silverstein, Nancy Morrow-Howell

## Abstract

The establishment of the Age-Friendly University (AFU) network and adoption of the 10 principles by institutions of higher education, was a major advance in the United Nations Sustainable Development Goal of promoting healthy and active aging through opportunities for intergenerational communities. AGHE endorsed the principles in 2016, since then over 60 institutions have joined the global network. Tools are needed to identify benchmarks that institutions can use to assess progress toward realizing the AFU principles on their own campuses. This symposium shares work done at the University of Massachusetts Boston, to develop and refine the AFU Inventory and Campus Climate Surveys (ICCS), a survey-based assessment instrument (developed from a prior pilot study in 2018) based on the premise that it is necessary to assess both the institution’s actual age-friendly practices and its perceived age-friendliness or campus climate. In August, 2019, the University of Massachusetts President’s office endorsed the 10 principles for the entire UMass system of 5 campuses, presenting an opportunity to assess a multi-campus system. To date, we have surveyed UMass Boston, UMass Lowell, UMass Dartmouth and UMass Medical (n=2,704). Testing and refinement of the AFU ICCS will contribute to both short- and long-term recommendations to assist in strategic planning by higher education institutions. Whitbourne will present the Inventory reporting tool. Bowen will present the Climate Survey. Gautam and Revell will describe the AFU work at UMass Lowell and UMass Dartmouth respectively and the use of the assessment tools on their campuses; Morrow-Howell will serve as Discussant.

